# Effect of Wu Zhi San supplementation in LPS-induced intestinal inflammation and barrier damage in broilers

**DOI:** 10.3389/fvets.2023.1234769

**Published:** 2023-12-04

**Authors:** Han Sun, Xirui Zheng, Bowen Yang, Mingen Yan, Huiting Wang, Shijing Yang, Dayou Shi, Shining Guo, Cui Liu

**Affiliations:** ^1^College of Veterinary Medicine, South China Agricultural University, Guangzhou, China; ^2^Guangdong Technology Research Center for Traditional Chinese Veterinary Medicine and Nature Medicine, Guangzhou, China; ^3^International Institute of Traditional Chinese Veterinary Medicine, Guangzhou, China

**Keywords:** LPS, traditional Chinese formula, intestinal inflammation, barrier damage, broiler

## Abstract

Intestinal inflammation and barrier damage can inhibit the absorption and transportation of nutrients in the small intestine, and lead to various chronic diseases. Wu Zhi San (WZS) is a traditional Chinese formula composed of Schisandrae, Anemarrhenae, Lonicerae, and Glycyrrhizae that was made to cure intestinal inflammation and barrier damage in broilers. To evaluate the protective effect of WZS on intestinal inflammation and barrier damage of broilers under lipopolysaccharide (LPS) stress, a total of 200 one-day-old broilers were randomly divided into five groups, namely, the CON group, LPS group, and three WZS groups (WZS-H, WZS-M, and WZS-L). The groups were designed for stress phase I (days 15, 17, 19, and 21) and stress phase II (days 29, 31, 33, and 35). The protective effect of WZS on the intestinal tract was evaluated by measuring the levels of serum myeloperoxidase (MPO), diamine oxidase (DAO), super oxide dismutase (SOD), and serum D-lactate (D-LA) and the expression of inflammatory factors in jejunum. The results showed that the diet supplemented with WZS could significantly reduce serum MPO, DAO, and D-LA levels and jejunal CD in broilers (*p* < 0.05), increase serum SOD levels and jejunal VH (*p* < 0.05), significantly downregulate the expression of NF-κB, TLR4, MyD88, and inflammatory cytokines (TNF-α, IL-1β, IL-6, and IL-10), and upregulate Claudin-1, Occludin-1, and ZO-1 in broiler jejunum mucosa (*p* < 0.05). On the other hand, WZS could significantly reduce the protein expression of NF-κB (p65) in broiler jejunum (*p* < 0.05). These results indicate that supplementing WZS in the diet can reduce intestinal inflammation and alleviate intestinal barrier damage, and by inhibiting the NF-κB/TLR4/MyD88 signaling pathway, supplementation with WZS intervenes in LPS-induced stress injury in broilers.

## 1 Introduction

Under the management of modern intensive farming modules, broilers are affected by various stress factors, such as environmental pressure, LPS, and pathogenic microorganisms, that can cause intestinal inflammation (1–3). The intestine serves as an immune barrier and the first line of defense against intestinal microbial infections, consisting of a mucus layer, tight intercellular junctions, antimicrobial peptides, and immunoglobulin A (4). It can prevent the invasion of exogenous pathogens or harmful substances, while also playing a crucial role in maintaining intestinal homeostasis and physical health (3, 5). When the mucus layer and intercellular tight junctions in the small intestine are disrupted, endotoxin produced by the metabolism of metabolizing bacteria enters and leaves the bloodstream, causing systemic and intestinal inflammation, disrupting the intestinal barrier, increasing intestinal permeability, inhibiting the absorption and transportation of nutrients in the small intestine, and leading to various chronic diseases (6, 7).

Intraperitoneal injection of LPS can lead to intestinal inflammation, increased intestinal permeability, and intestinal barrier damage. It has been reported that LPS challenge decreased mRNA abundances of β-defensin 2 (pBD-2), mucin (MUC-4), zona occludens 1 (ZO-1), and occludin in jejunal mucosa of piglets (8–10). LPS can recognize the cell surface transmembrane protein Toll-like receptor 4 (TLR4) and activates signaling pathways via myeloid differentiation factor 88(MyD88), which activates the nuclear factor-kappa B (NF-κB) signaling cascade. NF-κB can promote the production and release of inflammatory factors, thereby participating in inflammatory reactions and causing oxidative reactions in the body (11).

Research has shown that many traditional Chinese medicines can improve LPS-induced intestinal mucosal damage in broiler chickens and inhibit intestinal inflammation and oxidative stress, such as by inhibiting the expression of intestinal inflammation genes and intervening with Nrf2 and NF-κB pathways (12, 13). To address the problem of intestinal inflammation and barrier damage in broilers in poultry farming, we wove the formula of WZS. WZS is composed of Schisandra, Anemarrhenae, Lonicerae, and Glycyrrhizae, and has anti-inflammatory, antioxidant, and immunomodulatory effects. Schisandra has immunomodulatory effects, such as invigorating qi, invigorating fluid, and tonifying the kidney (14). Schisandra extract can ameliorate DSS-induced colitis, and its effect may be associated with suppression of the TLR4/NF-κB/NLRP3 inflammasome pathway and GM regulation (13). Research has shown that the active component of Anemarrhena asphodeloides B can block the production of NF-κB, and antipyretic effects are achieved by inhibiting the p38 mitogen-activated protein kinase pathway (15). In addition, Mangiferin in Anemarrhena has strong antioxidant effects (16). Lonicerin can target EZH2 to alleviate ulcerative colitis by autophagy-mediated NLRP3 inflammasome inactivation (17). Glycyrrhiza uralensis could regulate apoptosis of intestinal mucosal cells, through regulating the expression of apoptosis-related proteins and protective proteins of intestinal mucosa (18).

Therefore, this study investigated whether the addition of WZS to the diet could alleviate LPS-induced intestinal inflammation and barrier damage in broilers by inhibiting the inflammatory pathway and enhancing intestinal tight junction protein gene expression.

## 2 Materials and methods

### 2.1 Preparation of WZS

The traditional Chinese formula of WZS was composed of Schisandrae, Anemarrhenae, Lonicerae, and Glycyrrhizae in the ratio of 1:1.5:1.2:2, respectively. Schisandrae was purchased from HuaCong Pharmaceutical Co., LTD (HuaZhou, China). Anemarrhenae was purchased from China National Medicines Corporation Ltd. (Beijing, China). Lonicerae was purchased from Guangdong Shizhen Pharmaceutical Co (Guangzhou, China). Glycyrrhizae was purchased from Hebei Chu Feng Chinese Medicine Tablet Co. (AnGuo, China). The herbal materials of WZS were mixed and made into powder through a grinder, and their components and effects are listed in Table 1.

**Table 1:** The composition of WZS.

Latin name	Chinese name	Actions
Schisandrae	Wu Wei Zi	Enhancing Qi and astringing Yin. Calms the mind
Anemarrhenae	Zhi Mu	Antipyretic effect and promoting the production of bodily fluids
Lonicerae	Jin Yin Hua	Antipyretic and inflammatory effects and detoxifying the body
Glycyrrhizae	Gan Cao	Harmonizes the effects of other herbs

### 2.2 Reagents

Lipopolysaccharide (LPS, O55:B5) was purchased from Enzymax (China agent, Borealis Bio, Beijing). RNA Preservation Solution (202108) was purchased from Guangzhou Jiajie Biotechnology Co. (Guangzhou, China). Chicken D-LA ELISA KIT (202210) and Chicken DAO ELISA KIT (202210) were purchased from Guangzhou Jiajie Biotechnology Co. (Guangzhou, China). Superoxide Dismutase (SOD) assay kit (20220317) and Myeloperoxidase assay kit (20220308) were obtained from Nanjing Jiancheng Bioengineering Institute (Nanjing, China). RNA isolation Total RNA Extraction Reagent (017E2272CA) and Cham Q Universal SYBR qPCR Mix (027E2201CA) were purchased from Vazyme Biotech Co., Ltd. (Nanjing, China). NF-κB antibody (GR3309451-1) was purchased from Abcam Plc. (Shanghai, China). GAPDH antibody (GR3309451-1) was purchased from Proteintech Group, Inc. (Wuhan, China). A real-time fluorescence PCR system (qTOWER3G, Kepeng Scientific Instruments Co, Guangzhou, China) and a cDNA synthesis kit (R312-01/02. Vazyme Biotech Co., Ltd. Nanjing, China) were used. The primers were provided by Sangon Biotech Co., Ltd. (Shanghai, China).

### 2.3 Animal ethics statement

Broilers were provided by Enping Kilong Industrial Co., Ltd. (Jiangmen, China). Enping Kilong Industrial Co., Ltd., as a clinical practice cooperation base for experimental animals of South China Agricultural University, meets the basic requirements for Good Clinical Practice operations. All experimental procedures in this study were approved by the Animal Ethics Committee of the South China Agricultural University (Guangzhou, China).

### 2.4 Experimental design

A total of 200 one-day-old broilers were randomly divided into five groups. Three WZS groups were fed with 2, 1, and 0.5% of WZS (WZS-H, WZS-M, and WZS-L, respectively), and the CON and LPS groups were fed with normal feed during the experiment. The diets were purchased from Enping Kilong Industrial Co. The composition of the base diet is given in Supplementary Table S1.

**Figure 1:**
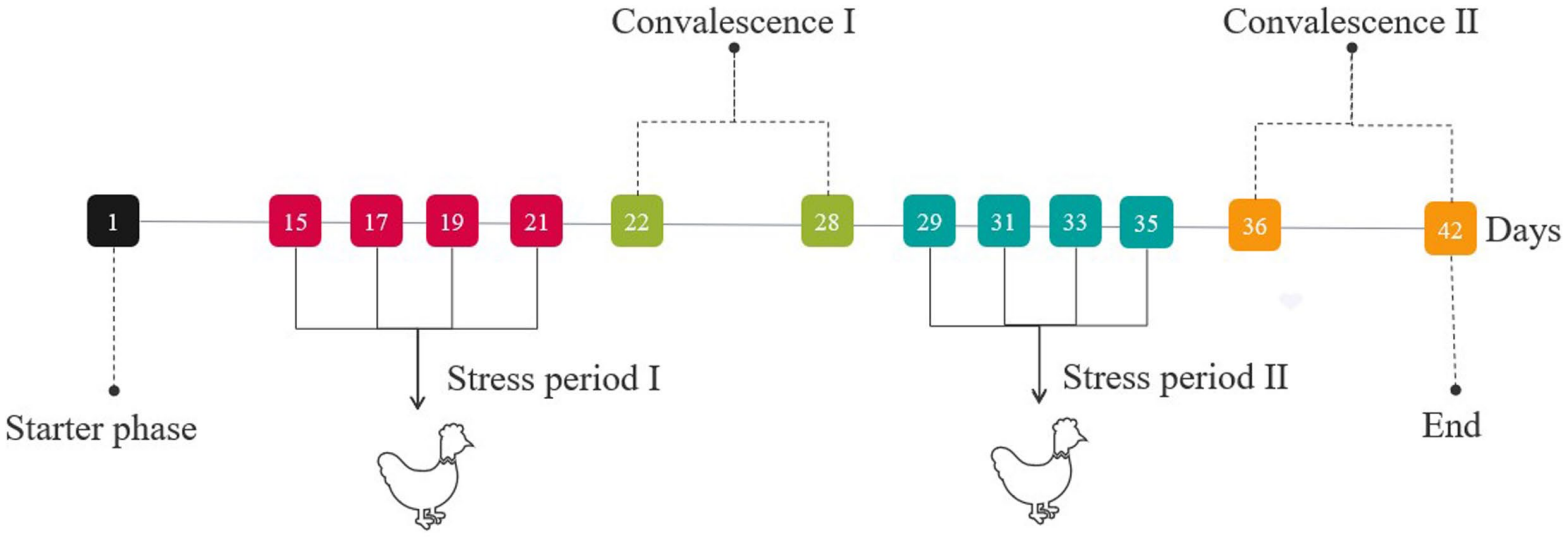
Treatment of the entire stage of the experiment.

During stress period I (on days 15, 17, 19, and 21) and stress period II (on days 29, 31, 33, and 35), broilers were injected intra-abdominally either with 500 μg/kg b.wt LPS solution, except the CON group (injected with an equal amount of sterile saline) (Figure 1). On days 21 (stress period I) and 35 (stress period II), cervical venous blood was collected. Then, the jejunum and hypothalamus tissues of broilers were collected immediately after euthanasia.

### 2.5 Determination of antioxidant indexes in chick serum

The serum supernatant was used to determine total SOD and MPO activities. Antioxidant indexes were measured by a Myeloperoxidase assay kit (Nanjing Jiancheng Technology Co., Ltd., 20,220,308) and a Superoxide Dismutase (SOD) assay kit (Nanjing Jiancheng Technology Co., Ltd., 20,220,317) according to the manufacturer’s instructions.

### 2.6 Intestinal permeability testing

The serum supernatant was used to determine antioxidant levels. Serum D-lactate (D-LA) and diamine oxidase (DAO) activities were measured using Chicken D-LA ELISA KIT and Chicken DAO ELISA KIT (Shanghai Enzyme-linked Biotechnology Co., Ltd., 202,210).

### 2.7 Morphological examination of the intestine

After fixing the jejunal tissue of each group of broiler chickens with formaldehyde for 1 week, paraffin embedding and hematoxylin and eosin (H&E) staining were performed (19). The intestinal tissue morphology was determined, and the villus height (VH) and crypt depth (CD) of the samples were measured to calculate the chorionic villus crypt ratio as follows:


$$\mathrm{Chorionic}\;\mathrm{villus}\;\mathrm{crypt}\;\mathrm{ratio}=VH\;\left(\mathrm{\mu m}\right)/CD\;\left(\mathrm{\mu m}\right)\text{.}$$

### 2.8 Quantitative PCR

Total RNA was extracted from the jejunum using a Trizol solution, and a performance cDNA synthesis kit for reverse transcription (20). Real-time fluorescence PCR was performed using Cham Q Universal SYBR qPCR mix and real-time fluorescence PCR system to detect NF-κB, MyD88, TLR4, TNF-ɑ, IL-1β, IL-10, IL-6, Claudin-1, Occudin-1, and ZO-1 gene expression. Transcript levels underwent relative quantification by the 2^−ΔΔCT^ method. All molecule expression was normalized against gene expression of specified housekeeping genes, namely β-actin. Primer sequence-related information of target genes is shown in Table 2.

**Table 2:** Primer sequences of target genes.

Gene name	Gene sequence number	Primer sequences
NF-κB	NM_001001472.3	F-GTGTGAAGAAACGGGAACTG R-GGCACGGTTGTCATAGATGG
MyD88	NM_001030962.5	F-GAGGATGGTGGTCGTCATT R-CATGGTCTTGCACTTGACCG
TLR4	NM_001030693.1	F-AGGCACCTGAGCTTTTCCTC R-TACCAACGTGAGGTTGAGCC
TNF-ɑ	NM_204267.2	F-CCTACCCTGTCCCACAACCT R-TGAACTGGGCGGTCATAGAA
IL-1β	NM_204524.2	F-CAGCCTCAGCGAAGAGACCTT R-ACTGTGGTGTGCTCAGAATCC
IL-10	NM_001004414.4	F-GCTGAGGGTGAAGTTTGAG R-CAGGTGAAGAAGCGGTGA
IL-6	HM179640.1	F-AAATCCCTCCTCGCCAATCT R-CCCTCACGGTCTTCTCCATAAA
Claudin-1	NM_001013611.2	F-TGGCCACGTCATGGTATGG R-AACGGGTGTGAAAGGGTCATAG
Occudin-1	NM_205128.1	F-ACGGCAGCACCTACCTCAA R-GGGCGAAGAAGCAGATGAG
ZO-1	NM_001265447.4	F-CCGCAGTCGTTCACGATCT R-GGAGAATGTCTGGAATGGTCTGA

### 2.9 Western blot analysis

The protein concentration in the jejunum was determined using the BCA protein assay kit. Adding an appropriate amount of lysate into jejunum tissue, the protein lysates were separated by 10% SDS-PAGE, transferred to PVDF membranes, and blocked with 5% skim milk powder for 1 h. Incubation with primary antibody NF-κB p65 and GADPDH was performed overnight at 4°C. At the end of primary antibody incubation, the secondary antibody was washed four times for 10 min with 1 × TBST and subsequently incubated for 2 h. Then, the band with a luminescent solution was detected.

### 2.10 Statistical analysis

SPSS22.0 software was used for statistical analysis of the data. One-way ANOVA was used for differences between groups, and Duncan’s multiple comparisons between groups were performed by Duncan’s multiple range test, with *p* < 0.05 representing statistical significance. GraphPad Prism 7 software was applied to draw graphs.

## 3 Results

### 3.1 Effect of WZS on antioxidant level in LPS-induced broilers

**Figure 2:**
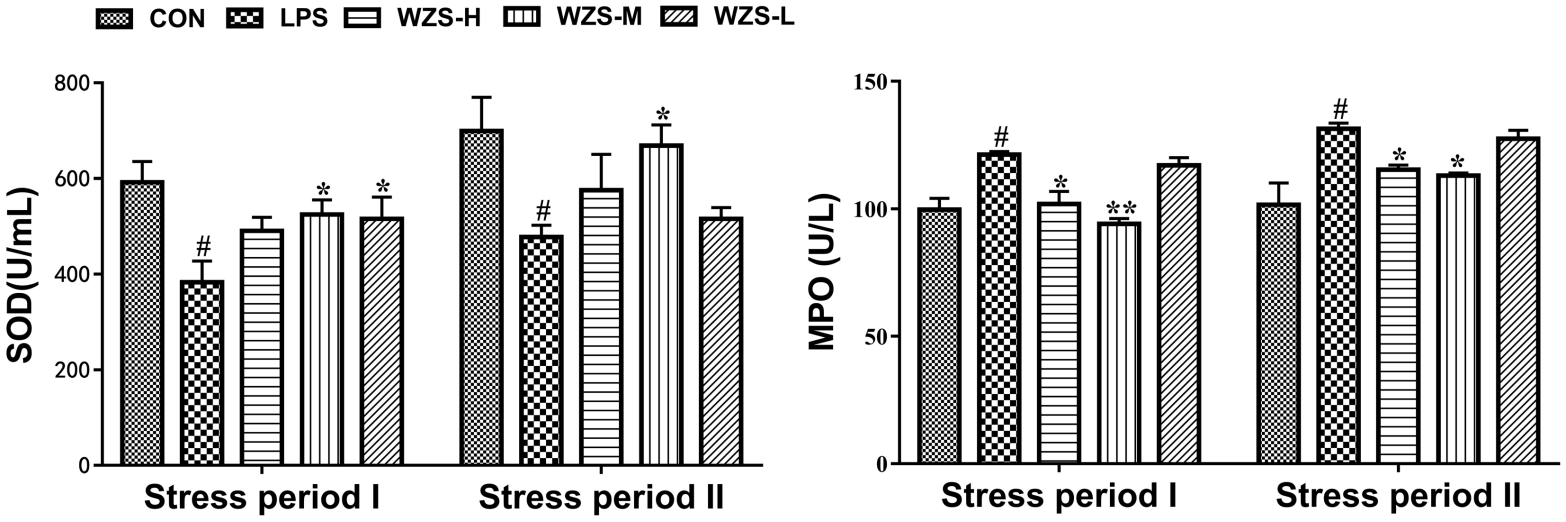
Changes in antioxidant indexes in the serum of broilers. ^#^ indicates a highly significant difference compared to the CON group (*p* < 0.05) and ^##^ indicates a highly significant difference compared to the CON group (*p* < 0.01). ^*^ indicates a significant difference compared to the LPS group (*p* < 0.05) and ^**^ indicates a significant difference compared to the LPS group (*p* < 0.01). The following graphs are the same.

During the stress period I, the SOD level in the serum of broilers in the LPS group was significantly reduced and the MPO level was significantly increased compared with the CON group (*p* < 0.05) (Figure 2). Compared with the LPS group, the MPO level in the serum of broilers in the WZS-H group was significantly reduced (*p* < 0.05), the SOD level in the serum of broilers in the WZS-M group was significantly increased, whereas the MPO level was highly significantly reduced, and the SOD level in the serum of broilers in the WZS-M group was significantly increased (*p* < 0.05).

During the stress period II, the SOD level in the serum of broilers in the LPS group was significantly reduced and the MPO level was significantly increased compared with the CON group (*p* < 0.05). Compared with the LPS group, the MPO activity in the serum of broilers in the WZS-H group was significantly reduced (*p* < 0.05), while the SOD level in the serum of broilers in the WZS-M group was significantly increased and the MPO level was significantly reduced (*p* < 0.05).

### 3.2 Effect of WZS on intestinal permeability index in LPS-induced broilers

**Figure 3:**
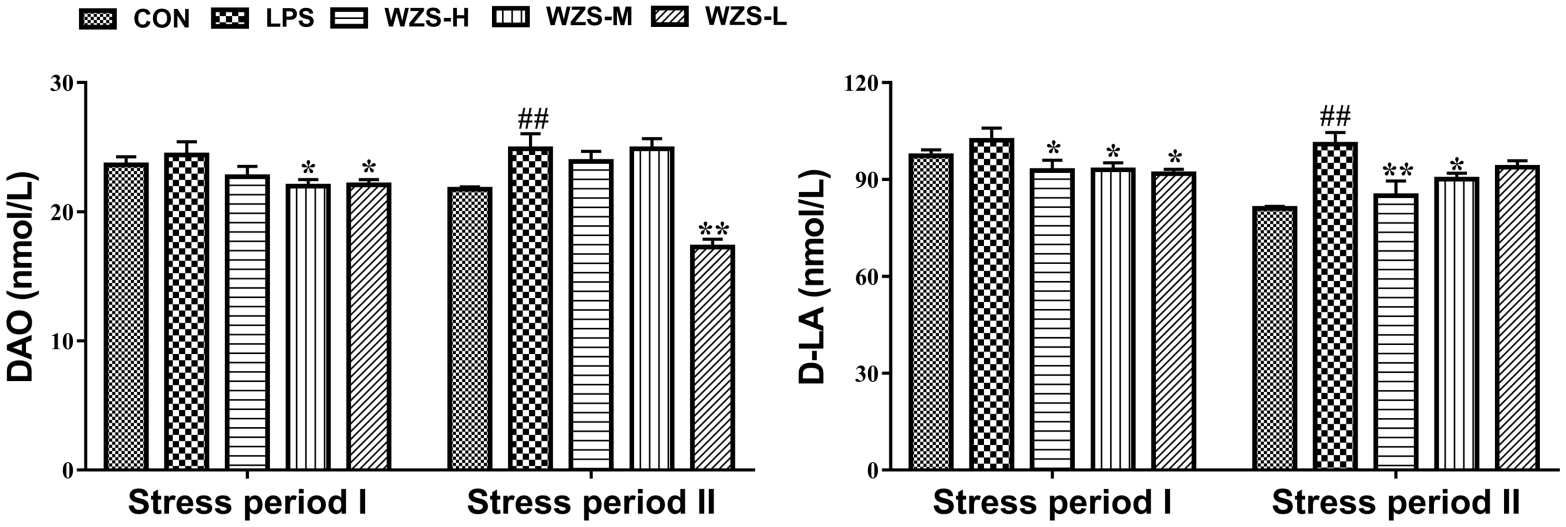
Changes in the intestinal permeability index of each group. ^#^ indicates a highly significant difference compared to the CON group (*p* < 0.05) and ^##^ indicates a highly significant difference compared to the CON group (*p* < 0.01). ^*^ indicates a significant difference compared to the LPS group (*p* < 0.05) and ^**^ indicates a significant difference compared to the LPS group (*p* < 0.01).

As shown in Figure 3, during the stress period I, the level of DAO and D-LA in the serum of broilers in the LPS group were not significantly different compared with the CON group (*p* > 0.05), but there was a tendency to increase. Compared with the LPS group, the level of D-LA in the serum of broilers in the WZS-H group was significantly reduced (*p* < 0.05), and the levels of both DAO and D-LA in the serum of broilers in the WZS-M and WZS-L groups were significantly reduced (*p* < 0.05).

During the stress period II, the level of DAO and D-LA in the serum of broilers in the LPS group were significantly increased compared with the CON group (*p* < 0.01), and the level of D-LA in the serum of broilers in the WZS-H group was significantly reduced (*p* < 0.01). Compared with the LPS group, the level of D-LA in the serum of broilers in the WZS-H and WZS-M groups was significantly increased (*p* < 0.05), and the level of DAO in the serum of broilers in the WZS-L group was significantly reduced (*p* < 0.01).

### 3.3 Effect of WZS on intestinal morphology of broilers

**Figure 4:**
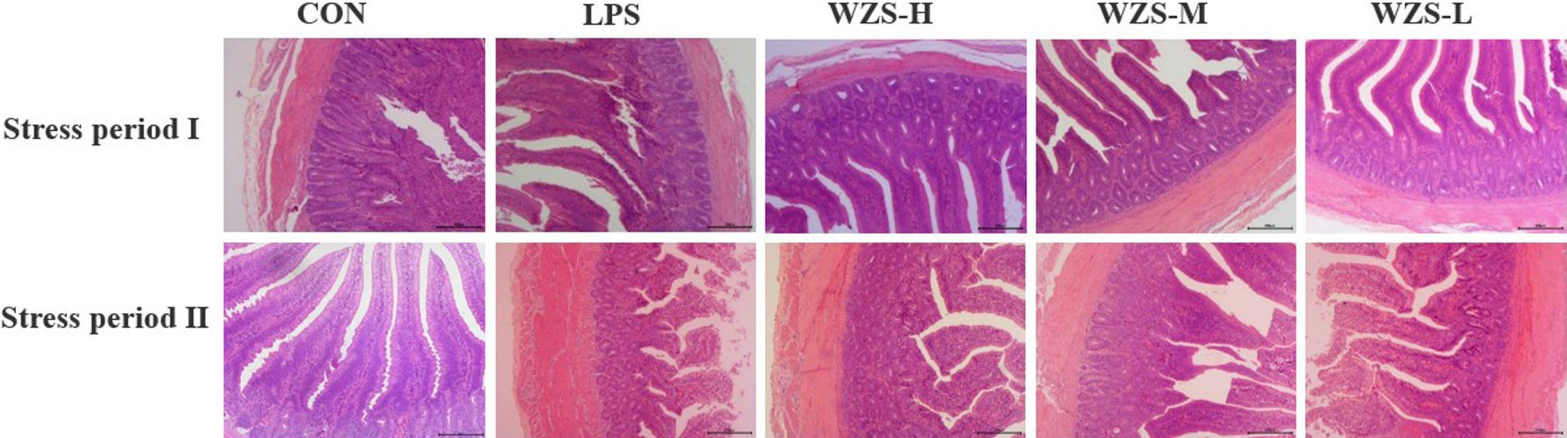
Histomorphological changes in jejunum tissues of each group.

Histopathology of jejunum tissues was observed under the light microscope (Figure 4). During the stress periods I and II, the broilers in the LPS group had significantly broken jejunum villi, increased gap, uneven arrangement, significantly reduced length, loss of intestinal epithelial cells, and increased inflammatory cell infiltration. The number of intestinal villi increased in each of the WZS groups, the length of the villi was greater than that of the LPS group, and the villi were neatly arranged.

As shown in Table 3, during the stress period I, broilers in the LPS group had a highly significant increase in jejunal CD and a significant decrease in VH/CD compared to the CON group (*p* < 0.01), and broilers in all dosing groups had a highly significant decrease in CD and a highly significant increase in both jejunal VH/CD compared to the LPS group (*p* < 0.05). During the stress period II, the LPS group showed a significant increase in CD in the jejunum of broilers, while both VH and VH/CD were significantly reduced compared with the CON group (*p* < 0.05). Compared with the LPS group, the jejunum VH and VH/CD of the broilers in the WZS-H group were highly significantly elevated, while the jejunum VH and VH/CD of the broilers in the WZS-M and WZS-L groups were highly significantly elevated (*p* < 0.05).

**Table 3:** Histomorphological changes in the jejunum.

Group	CON	LPS	WZS-H	WZS-M	WZS-L
Stress period I					
VH (μm)	908.39 ± 8.36	829.66 ± 32.90	973.61 ± 64.39^**^	1,063.22 ± 54.54^**^	1,157.00 ± 57.85^**^
CD (μm)	83.87 ± 4.14	104.91 ± 5.44^##^	52.67 ± 1.58^**^	64.98 ± 5.34^**^	69.32 ± 4.81^**^
VH/CD	10.85 ± 0.63	7.92 ± 0.41^#^	18.52 ± 1.79^**^	16.45 ± 1.88^**^	16.71 ± 0.41^**^
Stress period II					
VH (μm)	524.41 ± 63.73	391.95 ± 17.04^##^	754.24 ± 47.26^**^	869.00 ± 77.61^**^	908.83 ± 39.24^**^
CD (μm)	28.90 ± 7.46	60.86 ± 19.82^##^	56.84 ± 16.14	52.15 ± 2.81	38.40 ± 6.91^*^
VH/CD	18.67 ± 3.04	7.06 ± 2.90^##^	13.83 ± 3.10^*^	16.68 ± 1.56^**^	24.24 ± 4.75^**^

^#^ indicates a highly significant difference compared to the CON group (p < 0.05) and ^##^ indicates a highly significant difference compared to the CON group (p < 0.01). * indicates a significant difference compared to the LPS group (*p* < 0.05) and ** indicates a significant difference compared to the LPS group (*p* < 0.01).

### 3.4 mRNA expression levels of inflammatory factors in the jejunum

**Figure 5:**
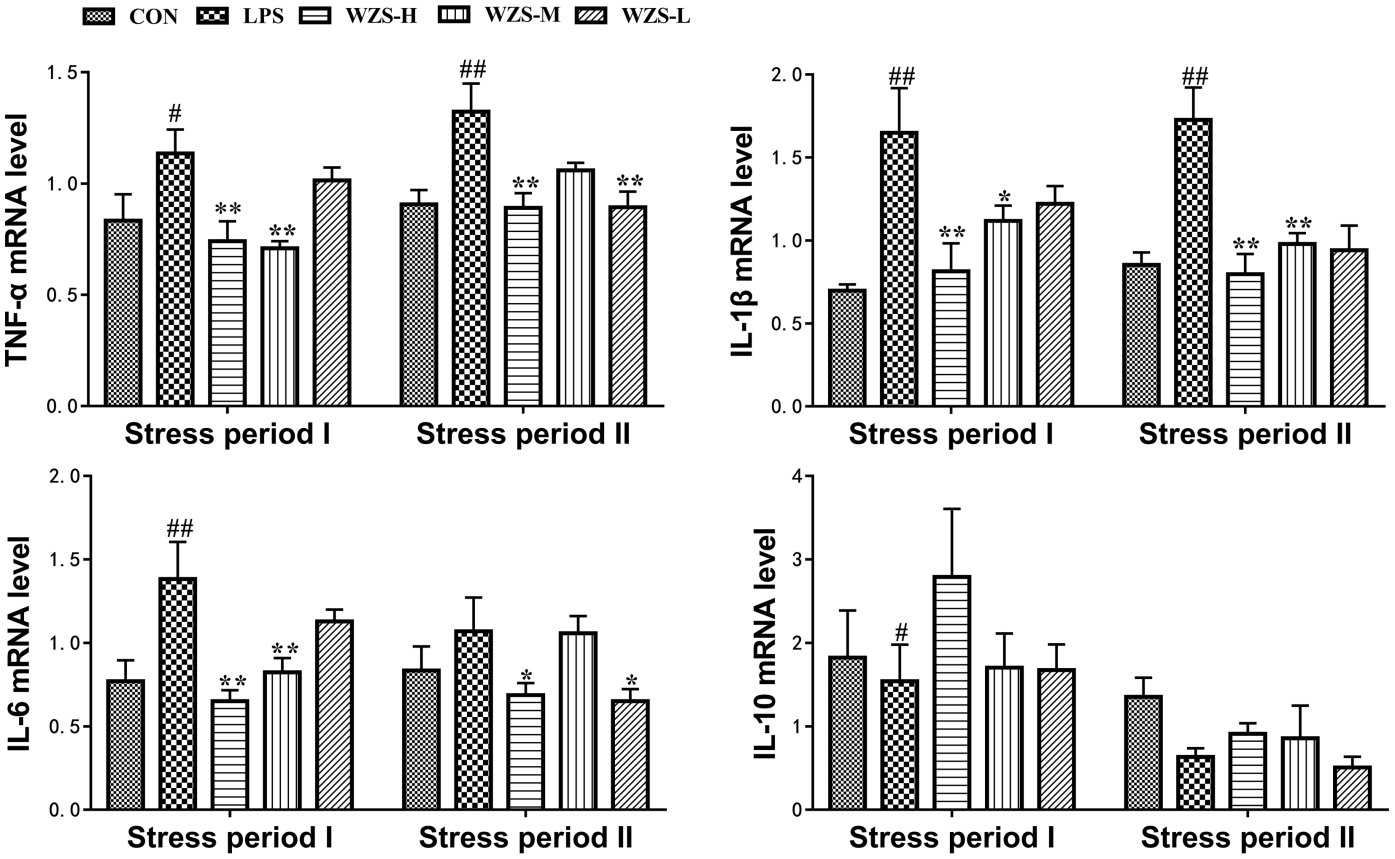
Changes in mRNA expression content of inflammatory factors in chick jejunum. ^#^ indicates a highly significant difference compared to the CON group (*p* < 0.05) and ^##^ indicates a highly significant difference compared to the CON group (*p* < 0.01). ^*^ indicates a significant difference compared to the LPS group (*p* < 0.05) and ^**^ indicates a significant difference compared to the LPS group (*p* < 0.01).

During the stress period I, the level of TNF-ɑ mRNA, IL-1β mRNA, and IL-6 mRNA in the LPS group was significantly increased in the jejunum of broilers compared with the CON group (*p* < 0.05, *p* < 0.01), and the expression of IL-10 mRNA was significantly reduced (*p* < 0.05) (Figure 5). Compared with the LPS group, the mRNA expression of TNF-ɑ, IL-1β, and IL-6 in the jejunum of broilers in the WZS-H group was highly significantly reduced (*p* < 0.01), the level of TNF-ɑ mRNA and IL-6 mRNA in the jejunum of broilers in the WZS-M group was highly significantly reduced (*p* < 0.01), the level of IL-1β mRNA was significantly reduced (*p* < 0.05), and the level of IL-10 mRNA of each dosing group showed an increasing trend.

During the stress period II, the level of TNF-ɑ mRNA and IL-1β mRNA in the jejunum of broilers in the LPS group was highly significantly increased compared with the CON group (*p* < 0.01). Compared with the LPS group, the mRNA expression of TNF-ɑ and IL-1β in the jejunum of broilers in the WZS-H group was significantly reduced (*p* < 0.01) and the mRNA expression of IL-6 was significantly reduced (*p* < 0.05). Furthermore, the level of IL-6 mRNA in the jejunum of broilers in the WZS-M group was significantly reduced (*p* < 0.01) and the level of TNF-ɑ mRNA in the jejunum of broilers in the WZS-L group was significantly reduced (*p* < 0.01). The expression of TNF-ɑ mRNA in the jejunum of broilers in the WZS-L group was significantly reduced (*p* < 0.01), and the level of IL-6 mRNA was also significantly reduced (*p* < 0.05).

### 3.5 mRNA expression of TLR4/ MyD88 / NF-κB in the jejunum of broilers

**Figure 6:**
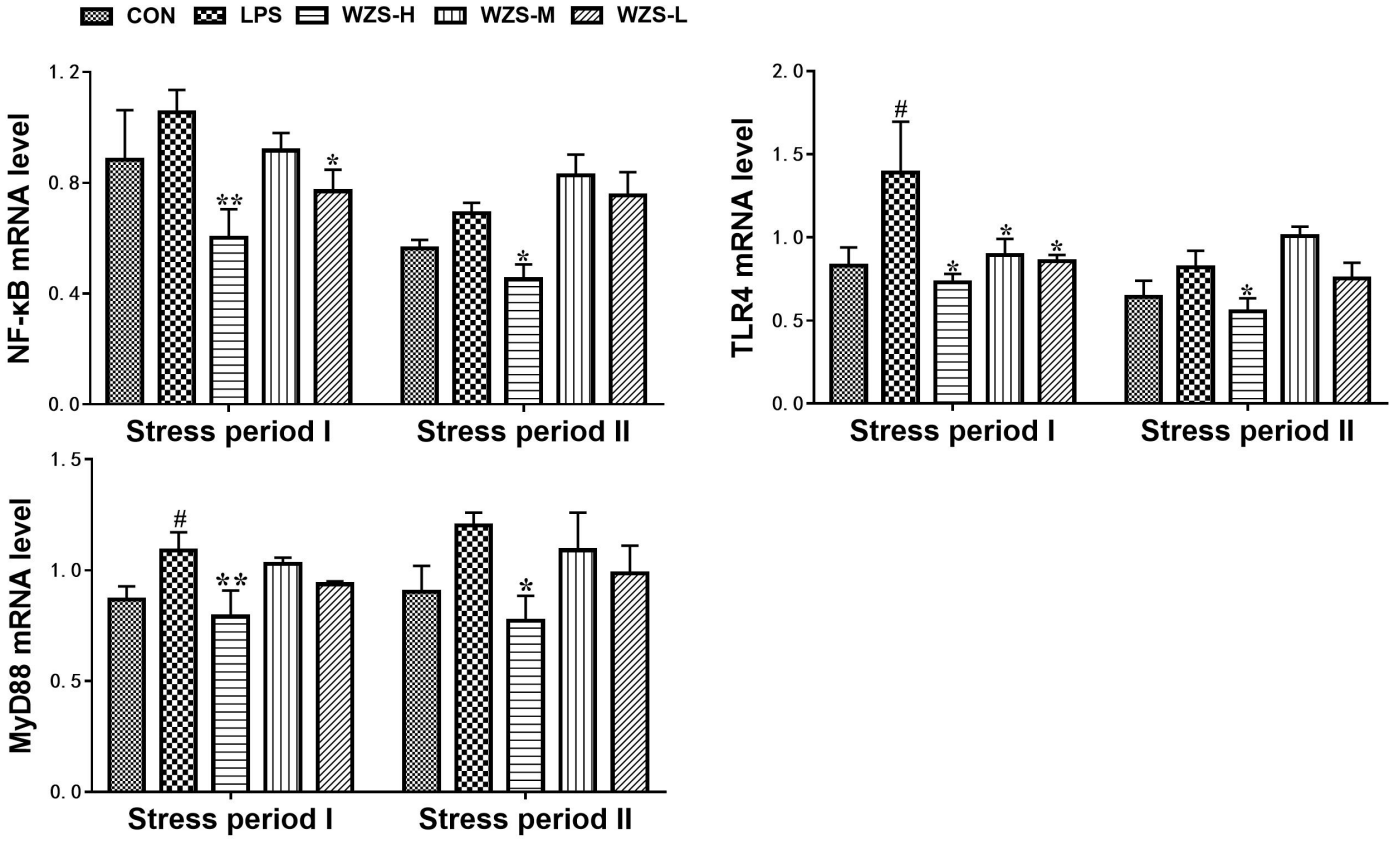
Changes in mRNA expression of inflammatory pathways in chick jejunum. ^#^ indicates a highly significant difference compared to the CON group (*p* < 0.05) and ^##^ indicates a highly significant difference compared to the CON group (*p* < 0.01). ^*^ indicates a significant difference compared to the LPS group (*p* < 0.05) and ^**^ indicates a significant difference compared to the LPS group (*p* < 0.01).

During the stress period I, the level of TLR4 mRNA and MyD88 mRNA in the jejunum of broilers in the LPS group was significantly increased compared to that of the CON group (*p* < 0.05) (Figure 6). During the stress period II, the mRNA expression of NF-κB, TLR4, and MyD88 in the jejunum of broilers in the LPS group was significantly increased compared with the CON group (*p* < 0.05), and the expression of TLR4 mRNA and MyD88 mRNA in the jejunum of broilers in the WZS-H group was significantly increased compared with the LPS group (*p* < 0.01). The mRNA expressions of NF-κB, TLR4, and MyD88 in the jejunum of broilers were significantly reduced in the WZS-H group compared with the LPS group (*p* < 0.05).

### 3.6 mRNA expression of tight junction genes in the jejunum of broilers

**Figure 7:**
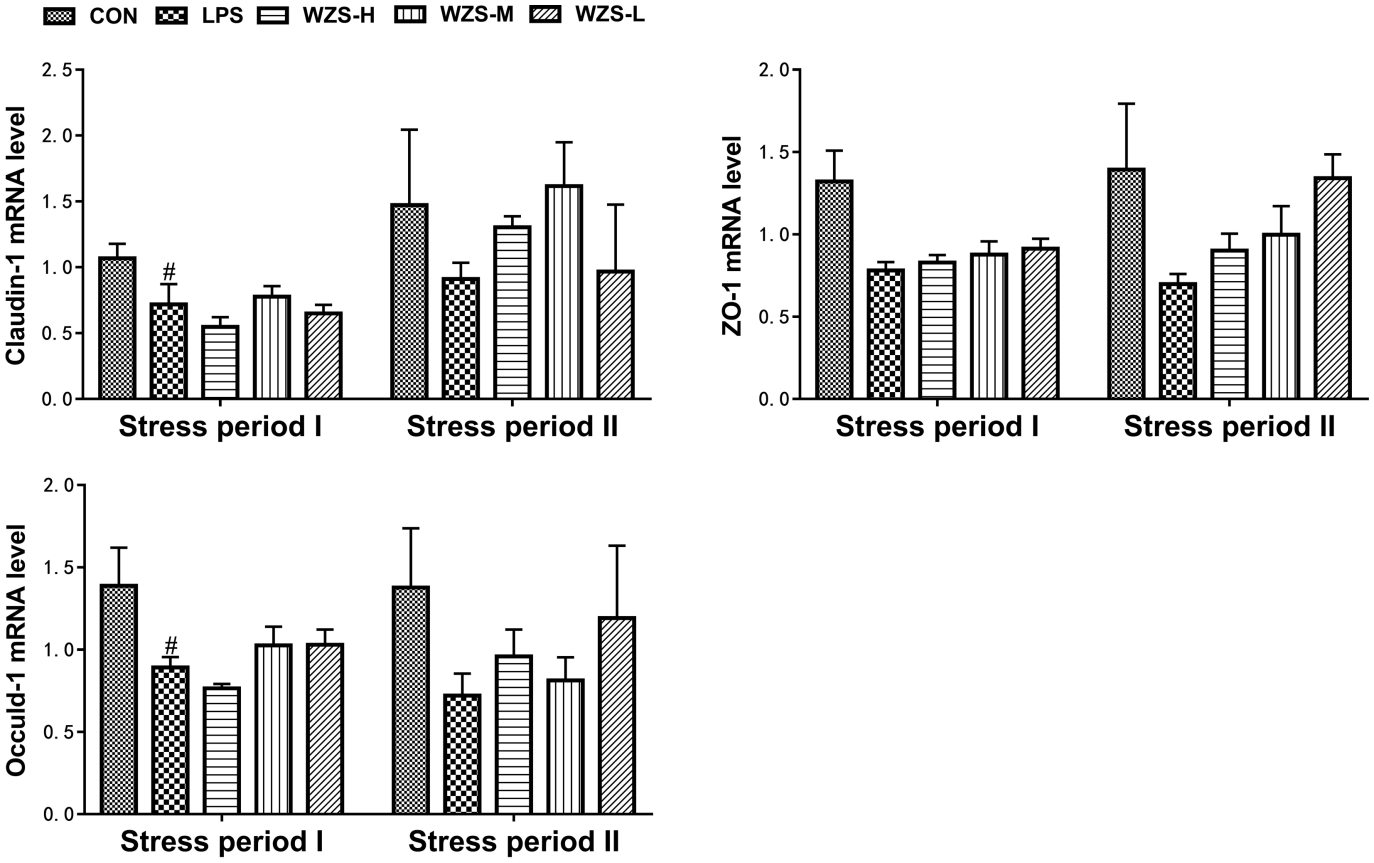
Changes in mRNA expression of chick jejunum tight junction protein. ^#^ indicates a highly significant difference compared to the CON group (*p* < 0.05) and ^##^ indicates a highly significant difference compared to the CON group (*p* < 0.01). ^*^ indicates a significant difference compared to the LPS group (*p* < 0.05) and ^**^ indicates a significant difference compared to the LPS group (*p* < 0.01).

During the stress period I, the mRNA expressions of Claudin-1 and Occuldin-1 in the jejunum of broilers in the LPS group were significantly decreased and the mRNA expression of ZO-1 was decreased compared with the CON group (*p* < 0.05). The mRNA expressions of ZO-1 and Occuldin-1 in the WZS-M and WZS-L groups had an increasing trend compared with the LPS group (Figure 7). During the stress period II, the mRNA expressions of Claudin-1, ZO-1, and Occuldin-1 in the jejunum of broilers in the LPS group were decreased compared with the CON group, and the mRNA expressions of Claudin-1, ZO-1, and Occuldin-1 in each administration group were increased to different degrees compared with the LPS group.

### 3.7 NF-κBp65 protein expression in the jejunum of broilers

**Figure 8:**
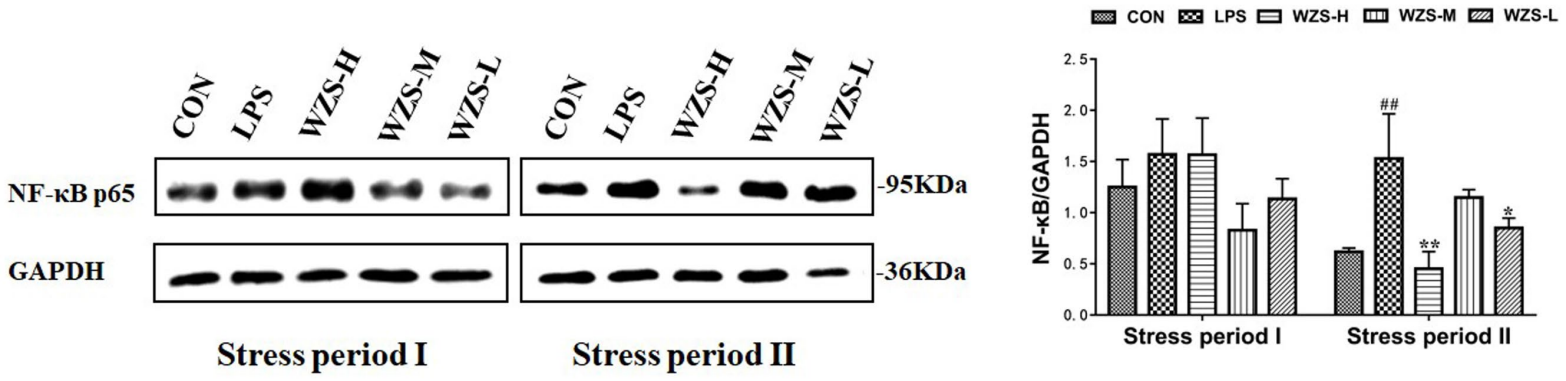
Changes in NF-κBp65 protein expression in chick jejunum. ^#^ indicates a highly significant difference compared to the CON group (*p* < 0.05) and ^##^ indicates a highly significant difference compared to the CON group (*p* < 0.01). ^*^ indicates a significant difference compared to the LPS group (*p* < 0.05) and ^**^ indicates a significant difference compared to the LPS group (*p* < 0.01).

There was no significant difference in jejunal NF-κBp65 protein expression among the groups compared to the CON group, but there was a significant decrease in the mid-dose group compared to the LPS group during the stress period I (Figure 8). The jejunal NF-κBp65 protein expression was highly significantly elevated in the LPS group compared with the CON group (*p* < 0.01), the jejunal NF-κBp65 protein expression was significantly decreased in the WZS-H group compared with the LPS group (*p* < 0.01), and the jejunal NF-κBp65 protein expression was highly significantly decreased in the WZS-L group compared with the LPS group (*p* < 0.05) during the stress period II.

## 4 Discussion

LPS activates macrophages in the animal body, leading to the production of large amounts of reactive oxygen species, which causes an imbalance in the production and elimination of excess reactive oxygen species in the body, resulting in lipid peroxidation in the body (21). As one of the products of lipid peroxidation, SOD activity can serve as a marker for evaluating the body’s antioxidant status. Its production is positively correlated with the content of oxygen free radicals, which can prevent the oxidation process initiated by superoxide anions and convert superoxide into hydrogen peroxide (22). MPO is a specific enzyme for neutrophils that reduces hydrogen peroxide and decreases the production of free radicals, and its level can be measured to directly reflect the number and activity of neutrophils (23). The more severe the inflammation, the higher the number of neutrophils and the higher the level of MPO (24, 25). The results showed that LPS could decrease SOD levels and increase MPO content in broilers, and all WZS groups could increase SOD levels in broiler serum to varying degrees, and effectively alleviate MPO content increase, among which the WZS-M group had the best effect. These results indicated that WZS had antioxidant and anti-inflammatory effects, and could significantly reduce the aggregation degree of neutrophils in chickens, therefore, reducing the inflammatory damage degree and oxidative stress damage degree of neutrophils to the body.

It has been shown that the levels of DAO and D-LA can reflect the damage of intestinal stress and are sensitive indicators that can evaluate intestinal permeability (26). LPS can damage the intestinal mucosal barrier in broilers, resulting in increased intestinal epithelial permeability (27). This is similar to the results of the present study. In this experiment, immune stress was able to damage the intestinal tract, leading to increased intestinal epithelial permeability and elevated serum DAO activity and D-LA levels. In both stress phases, all WZS groups were able to significantly reduce the serum levels of DAO and D-LA in broilers, indicating that WZS alleviated the intestinal epithelial damage caused by LPS by reducing the serum levels of DAO and D-LA in broilers.

The digestive and absorptive capacity of the intestine is usually reflected by VH, CD, and VH/CD indicators (28, 29). Mucosal integrity, digestive enzymes, transporter proteins, and intestinal microorganisms play an important role in the digestion and absorption of nutrients. Nutrient absorption occurs mainly in the small intestine, and to improve nutrient absorption in the small intestine, VH needs to be increased to expand the contact area between nutrients and the small intestine (30). It also requires a decrease in CD to promote the proliferation of intestinal epithelial cells, which further improves intestinal absorption. In the present study, under LPS stimulation treatment, broilers in the 21-day-old LPS group had significantly increased jejunal CD and significantly reduced VH/CD than the CON group, while the drug administration group was able to significantly reduce jejunal CD and increase VH/CD, among which the WZS-H group had the best effect. Broilers in the 35-day-old LPS group had significantly increased jejunal CD and significantly reduced VH and VH/CD when compared to the other groups. However, the WZS groups were able to alleviate this result to different degrees, and the effects were significant, among which the WZS-L group had the best effect. A study using fermented plant material added to broiler diets found that it significantly increased the villi length and villi length to crypt depth ratio of broiler jejunum under LPS stress, and significantly reduced the crypt depth of broiler jejunum (31). This is consistent with the results of the present study.

LPS binding to TLR4 activates the MyD88 pathway, which in turn induces nuclear displacement of NF-κB and induces gene expression of inflammatory factors, thereby activating lymphocytes to secrete anti-inflammatory cytokines to participate in the body’s immunity. If this signaling pathway is overactivated, the mRNA expression of NF-κB increases, which in turn causes an elevated expression of inflammatory factors leading to an inflammatory response (32). The results of the present study revealed that the mRNA expression of TLR4 in the jejunum of broilers in the LPS group was elevated to different degrees compared with the CON group in both stress phases, indicating that it was immune stress that led to the overexpression of TLR4 mRNA in the jejunum of broilers. Furthermore, the mRNA expression of MyD88 and NF-κB and the downstream signaling molecules of TLR4 were also significantly elevated and led to the protein amount of NF-κB. The expression of these genes, as well as NF-κB protein, was significantly increased in all administration groups, indicating that WZS can reduce the receptors necessary for LPS to attack cells, cut off the MyD88 pathway for LPS to invade cells, and reduce the overexpression of NF-κB genes and protein by decreasing the expression of TLR4 in chick jejunum. The expression in the WZS-L group showed the most obvious effect.

Overexpression of signaling molecules on the TLR4 signaling pathway leads to increased transcription of inflammatory factor genes, which in turn causes massive production of inflammatory factors in the organism. The test results showed that the mRNA expression of IL-6, IL-1β, and TNF-α in the jejunum of broilers in the LPS group increased significantly during the stress phase, indicating that immune stress leads to the overexpression of genes of inflammatory cytokines in the chick organism, resulting in an increase of inflammatory cells in the organism and causing damage to the organism. The mRNA expressions of IL-6, IL-1β, and TNF-ɑ in the jejunum of broilers in each administration group of this experiment were reduced to different degrees, indicating that the herbal compound can inhibit the gene expression of these inflammatory factors. Among them, the inhibition effect observed in the WZS-H group was better. This may be due to the fact that WZS can reduce the overexpression of related signaling molecules on the TLR4/MyD88/NF-κB signaling pathway, thus reducing the gene expression of inflammatory factors. It has also been shown that the increase of IL-1β disrupts the intestinal tight junctions and disrupts intestinal permeability (10). IL-10 could limit the activation of innate and adaptive immune cells to maintain homeostasis and protect the host from immune pathological damage, autoimmune damage, and allergic reactions induced by infection. During the stress period, IL-1β mRNA expression in the jejunum of broilers in the LPS group showed a significant increase, the expression of IL-10 mRNA in the jejunum of broilers in the LPS group was significantly reduced, and the mRNA expression of IL-10 in the jejunum of broilers in each WZS group was increased to different degrees, with the expression level of the WZS-H group being the highest and the effect being the best.

Tight junctions (TJs) are key components of the intestinal mucosal barrier and play an important role in regulating intestinal permeability by maintaining the integrity of the intestinal barrier and ensuring normal barrier function (33). ZO-1, Oclaudin-1, and white Claudin-1 are important components of TJs (34). In the present study, immune stress resulted in impaired jejunal tight junction structures characterized by a significant decrease in the mRNA expression of ZO-1, Occludin-1, and Claudin-1 in the jejunum of the LPS group, consistent with the results of previous studies (34, 35). The mRNA expression of Claudin-1, ZO-1, and Oclaudin-1 in the jejunum of broilers in each WZS group was elevated to varying degrees. This was due to the elevated intestinal permeability, which reduced the protective effect of the intestinal barrier on the intestine (36). Supplementing WZS in feed can alleviate the damage of LPS stress to the intestinal tight junction structure of broilers, and reduce the damage of inflammation to the body. However, the optimal dosage of WZS in feed needs to be further studied in subsequent experiments.

## 5 Conclusion

Dietary supplementation of WZS can reduce intestinal inflammation, reduce intestinal barrier damage, and intervene in LPS-induced stress damage by inhibiting the NF-κB/TLR4/MyD88 signaling pathway.

## Data availability statement

The original contributions presented in the study are included in the article/Supplementary material, further inquiries can be directed to the corresponding author.

## Ethics statement

The animal study was approved by the Animal Ethics Committee of the South China Agricultural University (Guangzhou, China). The study was conducted in accordance with the local legislation and institutional requirements.

## Author contributions

HS and CL conceived and designed the experiments. HS, BY, and SY completed the animal test. HS, XZ, and MY completed the laboratory test. HS and HW analyzed the data. HS and XZ wrote the original manuscript. HS, CL, XZ, HW, DS, and SG reviewed and edited the manuscript. CL supervised this study and provided funding. All authors contributed to the article and approved the submitted version.
